# Mutation Spectrum of Six Genes in Chinese Phenylketonuria Patients Obtained through Next-Generation Sequencing

**DOI:** 10.1371/journal.pone.0094100

**Published:** 2014-04-04

**Authors:** Ying Gu, Kangmo Lu, Guanghui Yang, Zhong Cen, Li Yu, Lin Lin, Jing Hao, Zhigang Yang, Jiabao Peng, Shujian Cui, Jian Huang

**Affiliations:** 1 Prenatal Diagnosis Center, Maternal and Child Health Care Hospital, Lianyungang, Jiangsu, China; 2 Prenatal Diagnosis Center, Chengdu Women's and Children's Central Hospital, Chengdu, Sichuan, China; 3 Shanghai Find Bio-Tech Co., Ltd., Shanghai, China; 4 Life Technologies Company, Shanghai, China; 5 National Engineering Center for Biochip at Shanghai, Shanghai, China; 6 Shanghai-MOST Key Laboratory for Disease and Health Genomics, Chinese National Human Genome Center at Shanghai, Shanghai, China; VU University Medical Center, Netherlands

## Abstract

**Background:**

The identification of gene variants plays an important role in the diagnosis of genetic diseases.

**Methodology/Principal Findings:**

To develop a rapid method for the diagnosis of phenylketonuria (PKU) and tetrahydrobiopterin (BH4) deficiency, we designed a multiplex, PCR-based primer panel to amplify all the exons and flanking regions (50 bp average) of six PKU-associated genes (*PAH, PTS, GCH1, QDPR, PCBD1* and *GFRP*). The Ion Torrent Personal Genome Machine (PGM) System was used to detect mutations in all the exons of these six genes. We tested 93 DNA samples from blood specimens from 35 patients and their parents (32 families) and 26 healthy adults. Using strict bioinformatic criteria, this sequencing data provided, on average, 99.14% coverage of the 39 exons at more than 70-fold mean depth of coverage. We found 23 previously documented variants in the *PAH* gene and six novel mutations in the *PAH* and *PTS* genes. A detailed analysis of the mutation spectrum of these patients is described in this study.

**Conclusions/Significance:**

These results were confirmed by Sanger sequencing. In conclusion, benchtop next-generation sequencing technology can be used to detect mutations in monogenic diseases and can detect both point mutations and indels with high sensitivity, fidelity and throughput at a lower cost than conventional methods in clinical applications.

## Introduction

Phenylketonuria (PKU), or hyperphenylalaninemia (HPA), is the most common metabolic disease in China due to autosomal recessive, inborn errors of phenylalanine metabolism. The overall incidence of PKU in China is 1/11,000 [1], [2], yet the prevalence varies considerably across China with much higher rates in northern China (1/3,425 - 1/7,849) than in southern China [3]–[7]. The worldwide regional and ethnic variation of PKU prevalence is also apparent, ranging from 1/100,000 in the African population to 1/2,600 in the Turkish population [8]. Patients with PKU usually present with HPA and severe mental retardation unless a low phenylalanine diet is introduced early in life. PKU can be classified as classic PKU, moderate PKU, mild PKU or mild HPA, depending on the enzyme defect, genotype and severity of the disease [8]. Genetic analysis has revealed that mutations in the *PAH* gene, which encodes phenylalanine hydroxylase, result in PKU. Thus far, more than 500 mutations have been deposited in the PAH mutation database and are comprised of 60.5% missense mutations, 13.5% deletions, 11% splice site mutations, 5% nonsense mutations and 1.8% insertions (http://www.pahdb.mcgill.ca/).

A deficit in tetrahydrobiopterin (BH4), the essential cofactor required for the hydroxylation of aromatic amino acids, may cause severe HPA and PKU, also known as malignant PKU, as well as rare neurological diseases with catecholamine and serotonin deficiencies with or without HPA. The estimated incidence of these deficiencies is reported to be 1–3% of all patients with HPA [9]. Unlike disease cases caused by a *PAH* mutation, patients with BH4 deficiency and HPA mainly have causal autosomal recessive mutations in the genes encoding the BH4 biosynthesis enzymes, including *PTS* (6-pyruvoyl-tetrahydropterin synthase) and *GCH1 (*GTP cyclohydrolase I), or the BH4 regeneration enzymes, including *PCBD1* (pterin-4a-carbinolamine dehydratase gene) and *QDPR* (dihydropteridine gene). Mutations are distributed across all the exons in each of these four genes without any identified hotspots [10]. GTP cyclohydrolase I feedback regulatory protein (GFRP) interacts with GCH1 in the liver [11]. Thus, GFRP may also play roles in phenylalanine metabolism, although no mutations have yet been found in this gene in humans.

Since next-generation sequencing (NGS) platforms first arrived on the market in 2005, their advantages over the classic Sanger sequencing method for genetic research have been demonstrated, including the ability not only to produce massive amounts of data in parallel but also to measure each base pair to an unprecedented depth. However, several weaknesses of NGS have prevented its application in clinical molecular labs, including the high cost, long sequencing time (2–3 days), high level of infrastructure complexity and poor sample scalability. Recently, a benchtop sequencer, the Ion Torrent Personal Genome Machine (PGM), has been developed to overcome most of these disadvantages. Using semiconductors and non-optical sequencing technology in the PGM, multiple genomic loci and samples can be sequenced on one chip in just two hours of machine running time [12]. Therefore, this technology has greatly reduced the time and cost of sequencing each sample at each locus. Furthermore, this technology has already been successfully applied to sequencing several cancer-related genes [13]–[15]. However, because this platform is rather novel for NGS, few publications have described its application in the mutational analysis of single gene disorders [16], [17].

In this study, we aimed to use this simple, fast, scalable and cost-effective PGM platform to study the mutational spectra of six genes in 35 Chinese patients with PKU. The development of methodology for data optimization and analysis and the advantages and disadvantages of this platform in monogenic mutational analysis are discussed in detail.

## Methods

### Patients

The cohort study consisted of 93 subjects from 32 families, including 33 patients diagnosed with PKU and two patients diagnosed with BH4 deficiency according to the diagnostic criteria published in Blau et al., 2011 [18]. Samples from 29 complete pedigrees, termed core families, and six incomplete pedigrees, termed non-core families, were available for this study. No consanguineous families were included in this study. The project and protocol for the investigation involving humans and animals were approved by the ethics committee of the Chinese National Human Genome Center at Shanghai. All patients provided their written informed consent to participate in this study according to the Declaration of Helsinki.

### DNA extraction, primer design and multiplex PCR

Genomic DNA was extracted from peripheral blood leukocytes using a QIAamp DNA Mini Kit (Qiagen). To directly sequence the 39 exons of *PAH, PTS, GCH1, QDPR, PCBD1* and *GFRP* genes, including 50 bp in the intron-exon boundaries, 108 pairs of primers were designed using the freely available software Primer Premier 5.0. Known variants were not included in the primer sequences (dbSNP build 138). Multiplex PCR was performed to reduce the number of amplification reactions. Each primer (at a final concentration of 10 μM) and 100 ng of genomic DNA were mixed together with 0.2 mM dNTPs and 10× buffer to a final reaction volume of 25 μl. Between 13 and 23 primer pairs with similar annealing temperatures were combined in one reaction, and the PCR amplification conditions varied for each multiplex PCR (95°C for 5 min followed by 30 cycles of 95°C for 30 s, the annealing temperature (Set 1: 58°C, Set 2: 56°C, and Set 3: 50°C) for 30 s and 72°C for 30 s. A final elongation step of 72°C for 5 min was added in each case. The resulting amplicons were then purified using Ampure Beads, and the concentration was measured using the Qubit dsDNA HS Assay kit (Life Technologies) and the fragment size was detected using Agilent 2100 Bioanalyzer. Amplicons were then mixed in equal amounts.

### Library construction and Ion Torrent sequencing

The template preparation was performed according to the Ion OneTouch System protocol (PNMAN0006957, Rev. 6.0) according to manufactory instructions. Briefly, an input concentration of one DNA template copy per Ion Sphere Particle (ISP) was added to the emulsion PCR master mix. The emulsion preparation was followed by DNA clonal amplification using the Ion OneTouch instrument (Life Technologies). The ISPs were then recovered, and the template-positive ISPs were enriched using Dynabeads MyOne Streptavidin C1 beads (Life Technologies) in the Ion OneTouch ES instrument (Life Technologies). ISP enrichment was qualified using the Ion Sphere quality control kit (Life Technologies) and a Qubit 2.0 fluorometer (Life Technologies). The complete ISP samples were loaded on either Chip 314 or Chip 316 and prepared for sequencing using the Ion PGM 200 sequencing kit protocol (PN4474246, Rev. D). The sequencing runs were performed using 520 flows for 130 cycles.

### Data analysis and variant prioritization pipeline

**Figure 1:**
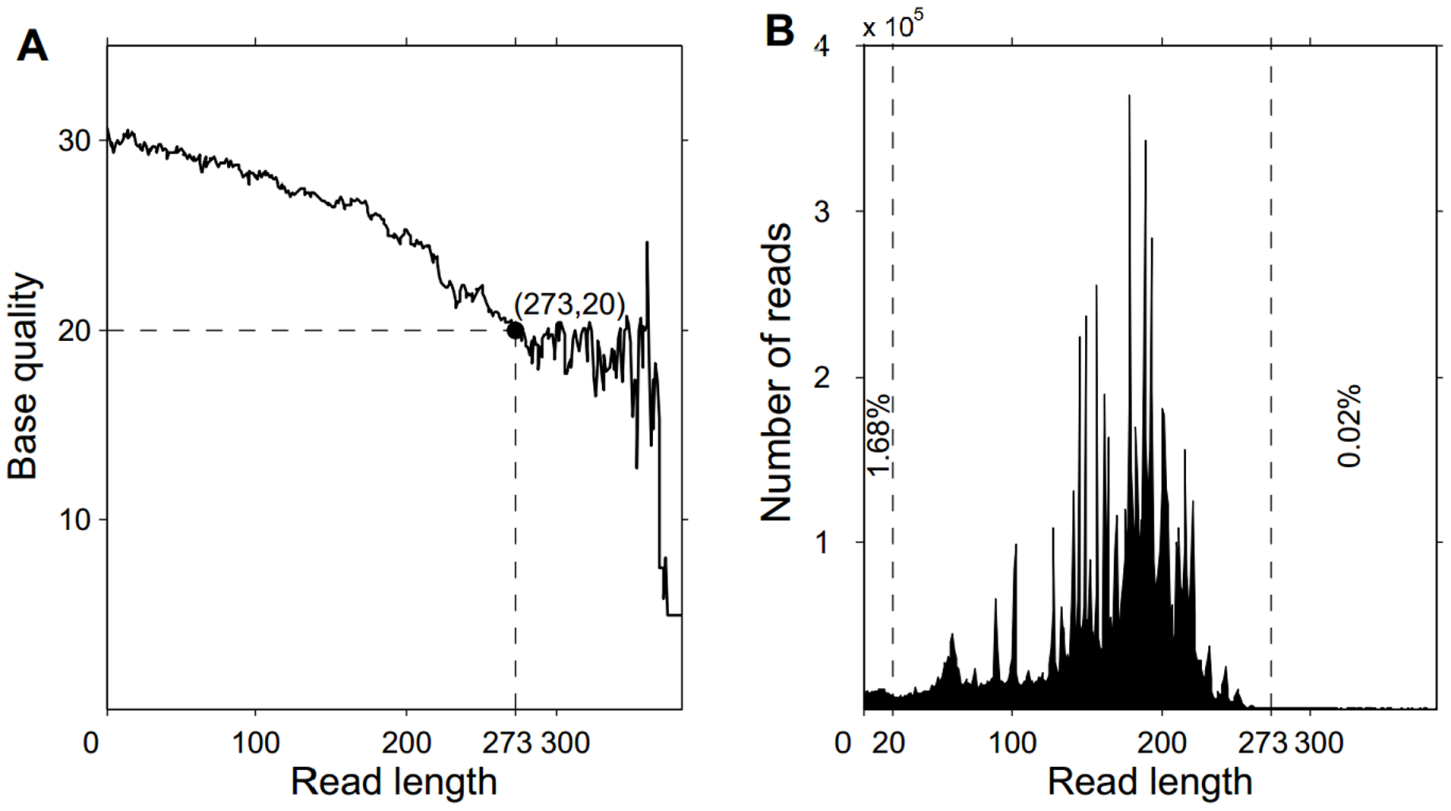
Selection criteria for trimmed reads. (A) The relationship between base quality and read length in the raw data. (B) The relationship between number of reads and read length in the raw data.

After the PGM run was complete, the data were processed using the Ion Torrent pipeline software Torrent Suite v3.4 (Life Technologies) to generate sequence reads. Barcode primers were used to identify the reads from different samples. After sorting, the adaptor sequences were removed from the reads. The sequence of each read was trimmed by 2 bp past the expected primer-indexed sequence length to accommodate a possible (or 99.7% likely) 2 bp insertion, because the 2 bp insertion has a 99.7% chance of occurring in our study. We further removed a few unqualified sequences from the primary data using a local dynamic programming algorithm, including very short reads of less than 20 bp (1.67% of the sequences, Figure 1) and reads with a quality score less than 20 (Figure 1), to increase the specificity of the read alignment. The remaining sequences were considered clean reads and were used for further analysis.

The clean reads were aligned against the human genome (HG19) reference sequence retrieved from the NCBI database (Build 37) using the BWA (Burrows Wheeler Aligner) Multi-Vision software package. Sequence variants, including indels, splice sites and single nucleotide variations (SNV), were identified using VarScan v2.3.5 software. The previously identified SNPs were determined using the NCBI dbSNP, HapMap or 1000 Human Genome Project databases. The well-known disease causing mutations were confirmed in the PAH mutations database (http://www.pahdb.mcgill.ca/), the Human Gene Mutation Database at the Institute of Medical Genetics in Cardiff (HGMD. http://www.ghmd.cf.ac.uk/) and previous publications.

All the sequence data (fastq) have been deposited in the NCBI SRA database; the accession number is SRR1171639 and the file name is “PKU research PGM data.”

### Sanger sequencing

The sample preparation and Sanger sequencing were performed by Life Technologies. Sanger sequencing was performed using the BigDye Terminator v1.1 Cycle Sequencing kit. After ethanol purification, the samples were run on an ABI 3730 sequencer. The chromatograms of the Sanger sequencing results were analyzed using SeqMan software.

## Results

### Multiplex PCR, high-throughput sequencing, alignment and coverage

To quickly discover DNA variants of the causal genes involved in genetic disorders and to sequence all the bases in the exons and intron-exon boundaries of the six genes (*PAH, PTS, GCH1, QDPR, PCBD1* and *GFRP*) involved in PKU and BH4 deficiency, 108 primer pairs were designed for multiplex PCR to amplify 39 exons containing 9,967 base pairs. The amplicons ranged in size from 103 to 267 bp (Table S1). To distinguish the sequences from different samples, we added a 10-bp barcode sequence to the 5′ end of each primer. After optimizing the PCR amplification conditions, we could use eight-time PCR reactions to amplify all the exons in each sample (Figure S1). The enriched DNA was used for library construction and then sequenced at single-base resolution using the Ion Torrent system. The total length of all PCR amplification products (PCR product + barcode sequence) from the six genes was approximately 22 kb (22,887 bp). In each sequence run, we obtained approximately 1.5 M reads (average read length was 200 bp) totaling 300 Mb of sequence using the Ion 316 chip. To obtain an average of 1,000-fold coverage of the targeted regions, 15 samples can be pooled in one sequence run using one Ion 316# chip.

**Figure 2:**
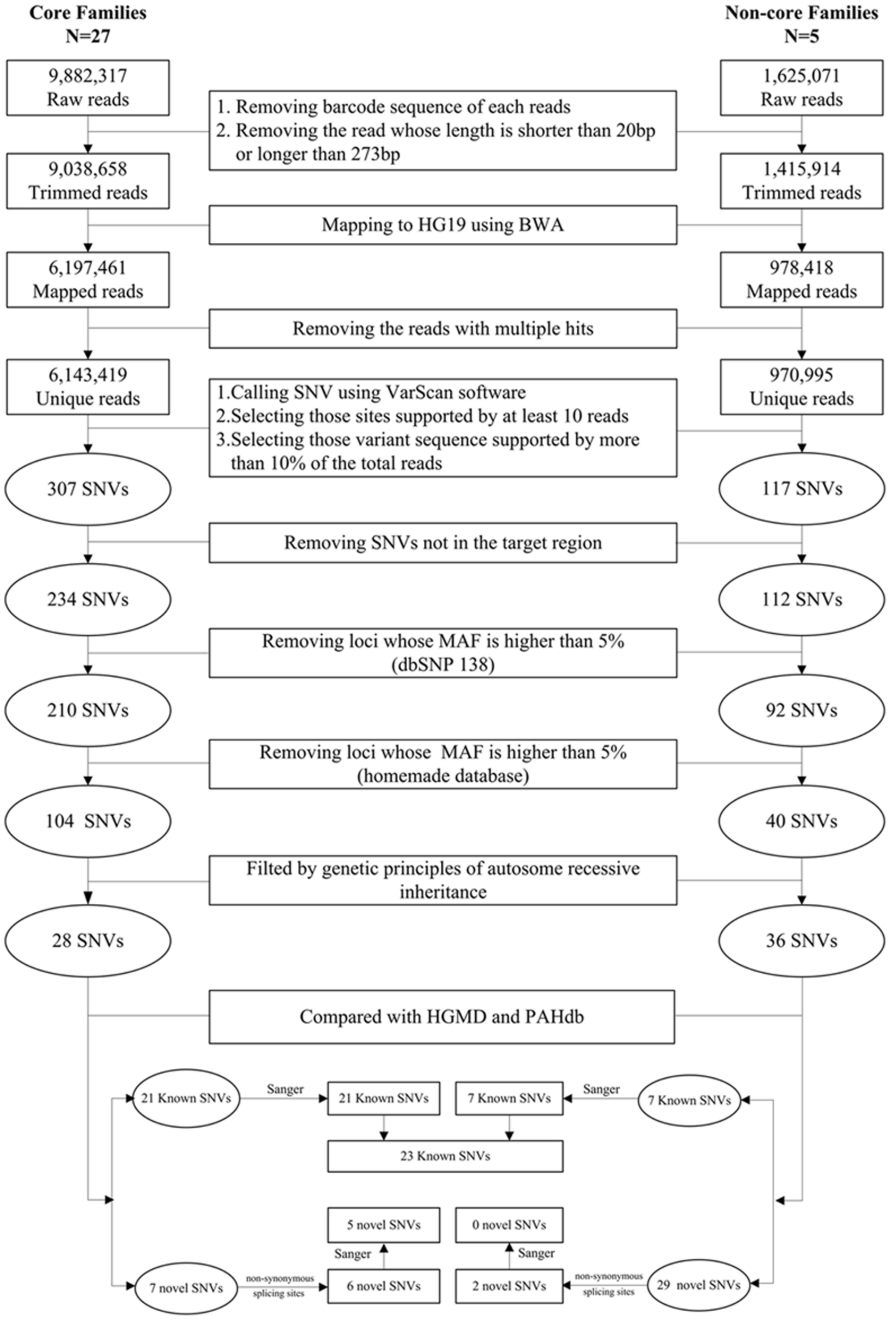
Flowchart for the process of screening and identifying single nucleotide variations (SNVs) in six genes by PGM sequencing.

Using the primer panel, we amplified all the exons in 93 samples from 35 PKU or BH4 deficiency patients and their parents (32 families) and 26 samples from healthy adults as controls. The PCR products were purified using magnetic beads (Agencourt NO.A63882, Beckman, Beverly, USA), and the enriched DNA from 15 samples was pooled together with same amount of each sample. After library construction, the pooled DNA library was sequenced using the Ion 316# chip. We obtained a total of 11,507,388 raw reads. To retain the high-quality reads for further analysis, we filtered all the raw data according to strict criteria (Figure 2) that removed the following: (1) the lower quality reads that were polluted by linker or adapter sequences; (2) the barcode sequence of each read; (3) those reads shorter than 20 bp (Figure 1A) because short reads are likely to align to multiple loci in the human genome; and (4) those reads longer than 273 bp because the majority of the bases after 273 bp had quality scores less than 20 (Figure 1B). (5) A final modification was performed because the reads obtained from the Ion Torrent sequencer are uneven in length; those reads longer than the expected amplicon size were cut to a length of the expected size plus 2 bp to analyze the potential insertion of 1 to 2 bp, which occurred in the overwhelming majority of reads in our study using the Ion Torrent system (Figure S2). For example, if the expected amplicon size was 103 bp, then the read was trimmed to 105 bp. After applying these filters, we obtained 8,420-375,137 high-qualitytrimmed reads per sample (Table S2). On average, 69.7% of the clean reads mapped uniquely to the human reference genome (HG19), with 93.9% of these reads mapping to the expected amplicon regions and 94.8% mapping to exon regions. We found that the average depth of coverage of all the 39 exons was 858.6-fold (Table S2) in 93 samples. The highest average depth of coverage was 3,069-fold in sample PKUD120003, and the lowest average depth of coverage was 74-fold in sample PKUD130082 (Table S2). Thus, the coverage and read depth should be adequate to reliably detect DNA variants within the majority of the targeted regions.

**Figure 3:**
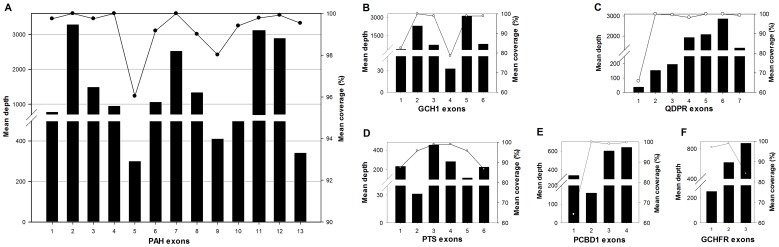
Mean depth of coverage and percentage of coverage of each exon of the six genes from all reads sequenced using a PGM. (A), (B), (C), (D), (E) and (F) indicate data from the ***PAH, GCH1, QDPR, PTS, PCBD1*** and ***GCHFR*** genes, respectively. The black columns indicate the mean depth of coverage, and the black circles indicate the mean percentage of coverage in each exon.

**Figure 4:**
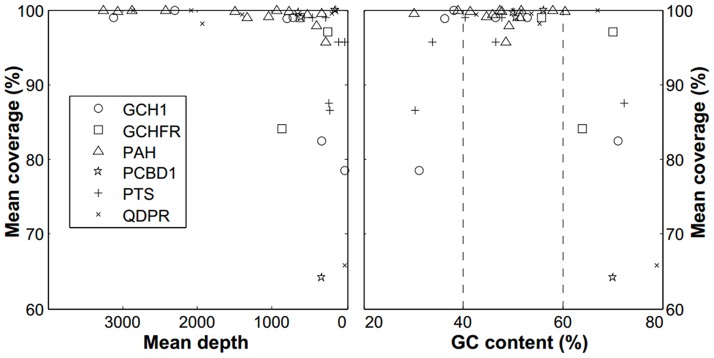
Comparison of GC content, read depth and coverage. The relationship between mean read depth and mean coverage (left) and the relationship between GC content and mean coverage (right) in each exon of the *PAH, GCH1, QDPR, PTS, PCBD1* and *GCHFR* genes. Exons with low coverage have relatively low read depth and excessively low (less than 40%) or high (more than 60%) GC content.

To evaluate the enrichment efficiency of the multiplex PCR, we analyzed the relationship between the mean percentage of coverage and mean depth of coverage of unique reads matched onto the six genes in all 93 samples using sequencing. Our results showed that the mean percentage of coverage and mean depth of coverage at the *PAH* gene (13 exons) in these samples was 99.3% and 1,466-fold, respectively (Figure 3A and Table S3). We also found that the mean percentage of coverage and mean depth of coverage at the other genes, including *GCH1* (6 exons) and *QDPR* (7 exons), were more than 93% and 1,200-fold, respectively (Figure 3B and 3C, Table S3), while the mean coverage and depth at the *PTS* (6 exons), *PCBD1* (4 exons) and *GFRP* (3 exons) genes were more than 90% and 200-fold, respectively (Figure 3D–3F, Table S3). However, we found that exon 4 in the *GCH1* gene and exon 1 in the *QDPR* gene had lower mean percentage of coverage (78.5% and 65.8%) and mean depth of coverage (32.7-fold and 36.9-fold), while exon 1 in the *PCBD1* gene had lower mean percentage of coverage (64.2%) and higher mean depth of coverage (340-fold) than the other exons. To explain these results, we analyzed the GC content of the 39 exons and evaluated the relationships between the GC content, mean percentage of coverage and mean depth of coverage. The findings showed that the exons with lower percentage of coverage (less than 90%) had lower (less than 40%) or higher (more than 60%) GC content, suggesting that the lower percentage of coverage was associated with excessively high or low GC content that can affect amplification and sequencing efficiency (Figure 4).

### Identification of DNA variant sites

To identify the variants in the six genes in each sample, the high-quality, unique reads were mapped to HG19 using BWA software, and the variants were called using VarScan software [19] according to the following procedure (Figure 2): (1) exclusion of sites not in the target regions, (2) selection of sites supported by at least 10 reads, (3) classification of heterozygous sites as those supported by 10–90% of the total reads at that site, with all other sites classified as homozygous and (4) selection of the loci whose Minor Allele Frequency (MAF) is less than 5% according to the dbSNP database or an in-house database from the 26 normal adults sequenced using the Ion Torrent system. Following this procedure, we identified 117 single-nucleotide variants (SNVs), of which 104 were from the 27 core families, 40 were from the five non-core families, and 27 were found in both groups (Figure 2).

### Identification of disease-causing substitution mutations for the diagnosis of PKU

To identify disease-causing mutations using the genetic principles of autosomal recessive inheritance (AR), we first selected those homozygous variant sites in patient samples that differed from the parental genotypes (Table S4). We then selected those heterozygous variant sites in the patient samples that represented one paternal allele and one maternal allele (Table S4). In our study, the majority of identified mutations fit into one of these two categories. After applying these selection criteria, we obtained 59 potential substitution mutations, of which 29 were from the 27 core families, 36 were from the five non-core families, and five were found in both groups (Figure 2, Table S4).

Next, these potential mutations were compared with the known disease-causing mutations deposited in disease databases, including the Human Gene Mutation Database (HGMD) [http://www.biobase-international.com/product/hgmd] and the mutation database for the human phenylalanine hydroxylase gene (PAHdb) [http://www.mcgill.ca/pahdb]. The 29 SNVs identified from the 27 core families included 21 known and 8 novel mutations, while the 36 SNVs identified from the five non-core families included seven known and 26 novel mutations (Figure 2, Table S4). From these novel potential mutations, we selected those sites with non-synonymous variants or proximity to splice sites. We finally obtained eight novel potential mutations, of which six were from the core families and two were from the non-core families (Figure 2, Table S4).

Furthermore, to validate the variants identified by NGS, we amplified all 31 candidate sites with potential mutations, including 23 known (21 from core family and 7 from non-core family) and 8 novel sites (6 from core family and 2 from non-core family), and their flanking sequences using the same primers used in NGS (Figure 2). We then sequenced the PCR products by Sanger sequencing using an ABI 3730 DNA Analyzer (Applied Biosystems). The results confirmed 23 (100%) of the 23 known mutations and five (62.5%) of the 8 novel mutations. The three SNVs that were not validated had a variant allele frequency of approximately 10%, suggesting that the Ion Torrent method used for mutation analysis provides high accuracy and a very low false-positive rate (Table S4).

**Figure 5:**
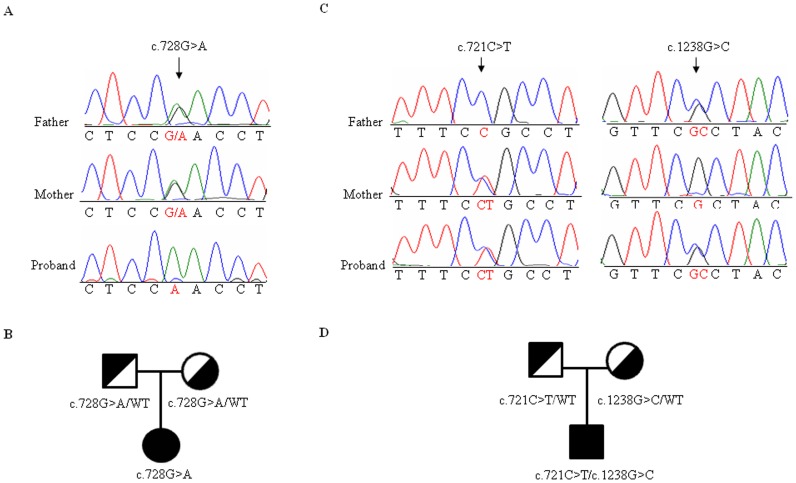
The representative mutation types observed based on the genetic principles of autosomal recessive inheritance. (A) A homozygous missense mutation of G to A in exon 7 of the PAH gene. The parents of this patient were confirmed to be heterozygous carriers for this mutation. (B) The family pedigree of the patient described in panel A. (C) A heterozygous missense mutation of T to A in exon 7 and a heterozygous mutation of G to A in exon 12 of the *PAH* gene. The father of this patient was a heterozygous carrier with a C allele in exon 12, while the mother was a heterozygous carrier with a T allele in exon 7. (D) The family pedigree of the patient described in panel C.

In the current study, we found one homozygous mutation in exon 7 of the PAH gene in sample PKUD120001 from F1 (Table S5, Figure 5A and 5B). In addition, the majority of identified mutations were found in heterozygous variants in this study, of which one was the paternal allele while the other was the maternal allele. The representation result was displayed in Figure 5C and 5D.Four validated novel mutations present in heterozygous variants included two missense mutations (c.875C>T and c.971T>A) (Figure S3A, S3B and Table 1), one frameshift deletion (c.669delC) (Figure S3C and Table 1) and one substitution at the intron 10 splice site (IVS10-13T>G) (Figure S3D and Table 1). To predict the phenotypic effects of the mutations at the protein level, evolutionary conservation analysis was performed and revealed that the two novel missense variants (c.875C>T and c.971T>A) were highly conserved across species, especially in mammals (Figure S4). Furthermore, these two non-synonymous substitutions (c.875C>T and c.971T>A) were predicted by to be damaging mutations (SIFT score = 0) by SIFT v2.0 (http://blocks.fhcrc.org/sift/SIFT.html) according to the standard which the score of the damaging substitution of amino acid predicted by SIFT is ≤0.05. These results showed that the detected novel mutations may play a role in the progression of the disease.

**Table 1:** Spectrum of PAH and PTS mutations.

Gene	ID	Variants		Locations	Characters	Type	Number of	Frequency	Family
		cDNA	Protein		of mutation		alleles	(%)	
PTS	1	c.259C>T	p.P87S	Exon 5	Missense	Known	2	3.28	Core
PTS	2	c.272A>G	p.K91R*	Exon 5	Missense	Novel	1	1.64	Core
PAH	3	c.208_210delTCT	p.S70del	Exon 3	Deletion	Known	1	1.64	Core
PAH	4	c.284_286delTCA	p.I95del	Exon 3	Deletion	Known	1	1.64	Core
PAH	5	c.442-1G>A	IVS4-1G>A	Intron 4	Splicing	Known	1	1.64	Core
PAH	6	c.482T>C	p.F161S	Exon 5	Missense	Known	2	3.28	Core
PAH	7	c.498C>A	p.Y166X	Exon 5	Nonsense	Known	1	1.64	Core
PAH	8	c.611A>G	p.Y204C	Exon 6	Missense	Known	3	4.92	Core
PAH	9	c.669delC	p.N223Kfs	Exon 6	Deletion	Novel	1	1.64	Core
PAH	10	c.699C>A	p.F233L	Exon 6	Missense	Known	1	1.64	Core
PAH	11	c.716G>A	p.G239D	Exon 7	Missense	Known	1	1.64	non-core
PAH	12	c.721C>T	p.R241C	Exon 7	Missense	Known	6	9.84	Core/non-core
PAH	13	c.727C>T	p.R243X	Exon 7	Nonsense	Known	1	1.64	Core
PAH	14	c.728G>A	p.R243Q	Exon 7	Missense	Known	10	16.39	Core/non-core
PAH	15	c.740G>T	p.G247V	Exon 7	Missense	Known	1	1.64	non-core
PAH	16	c.823C>T	p.P275S	Exon 7	Missense	Known	1	1.64	Core
PAH	17	c.833C>A	p.T278N	Exon 7	Missense	Known	1	1.64	Core
PAH	18	c.842+1G>A	IVS7+1G>A	Intron 7	Splicing	Known	1	1.64	Core
PAH	19	c.875C>T	p.P292L*	Exon 8	Missense	Novel	1	1.64	Core
PAH	20	c.971T>A	p.I324N*	Exon 10	Missense	Novel	1	1.64	Core
PAH	21	c.977G>A	p.W326X	Exon 10	Nonsense	Known	1	1.64	Core
PAH	22	c.1006C>T	p.Q336X	Exon 10	Nonsense	Known	1	1.64	Core
PAH	23	c.1045T>G	p.S349A	Exon 10	Missense	Known	2	3.28	Core
PAH	24	c.1066-11G>A	IVS10-11G>A	Intron 10	Splicing	Known	1	1.64	Core
PAH	25	c.1066-13T>G	IVS10-13T>G*	Intron 10	Splicing	Novel	1	1.64	Core
PAH	26	c.1068C>A	p.Y356X	Exon 11	Nonsense	Known	5	8.20	Core/non-core
PAH	27	c.1197A>T	p.V399V	Exon 11	Splicing	Known	4	6.56	Core/non-core
PAH	28	c.1222C>T	p.R408W	Exon 12	Missense	Known	1	1.64	Core
PAH	29	c.1238G>C	p.R413P	Exon 12	Missense	Known	4	6.56	Core/non-core
PAH	30	c.1242C>A	p.Y414X*	Exon 12	Nonsense	Novel	1	1.64	Core
PAH	31	c.1301C>A	p.A434D	Exon 12	Missense	Known	2	3.28	Core
Total							61	100.00	

* indicates stop code.

### Identifying disease-causing indel mutations

Given that the short insertions and deletions (indel) analysis is more difficult than variant identification; we integrated the results obtained by the two different algorithms. Firstly, all of the uniquely reads mapped to HG19 using BWA were used for calling indels using VarScan software. This analysis uncovered 498 indels, including 493 from the core families and 205 from the non-core families (Figure S5 left). In addition, the raw data were also analyzed using TMAP v3.4.1 and VariantCaller v3.4.5 software. This analysis identified 53 potential indels (Figure S5 right). We selected those loci that localized to coding sequences or splice sites and then we obtained 147 and 14 potential indels by BWA-VarScan and TMAP- VariantCaller, respectively. This selection process led to the identification of six potential indels. These six indels were validated by PCR-based Sanger sequencing, and three of them were confirmed (Figure S5 and Table S5).

## Discussion

Since arriving on the market in 2011, the Ion Torrent PGM, a benchtop sequencer, has already been successfully used for the targeted resequencing of cancer genes, such as *BRCA* [15], *BRAF* [14], *POLE* and *POLD1* [13]. However, only limited research has been conducted using this semiconductor sequencing platform in monogenic disease studies [16], [17]. To date, more than 7,000 single gene disorders have been identified in humans, and PKU is the most common inborn error of metabolism disease in China and is caused by mutations in the *PAH*, *PTS*, *GCH1*, *QDPR*, *PCBD1* and *GFRP* genes [20]. In this study, our aim was to construct a method for the rapid detection of all the nucleotide mutations in these six genes causing PKU disease. Together, 39 exons of these six genes were sequenced in 35 patients with either PKU or BH4 deficiency using the PGM system. After stringent read-filtering and the optimization of substitution and indel detection, 23 known substitution mutations in the *PAH* gene and five novel mutations in the *PAH* and *PTS* genes were identified. All of these mutations were confirmed by Sanger sequencing.

High-quality reads were selected in a strict manner to ensure quality results. When weak signals (quality scores less than 15) were detected during the sequencing step, the extension of that read was stopped. A weak signal could be due to problems in the emulsion PCR, such as one watery drop within the water-oil emulsion containing several Ion Sphere Particals (ISP) or bad amplification in one ISP. When the raw data were analyzed, these short reads often mapped to multiple positions. In this study, we removed those reads less than 20 bp to increase the alignment efficiency (Figure 1B). In addition, we found some reads with lower quality base pairs (scores less than 20) at positions past 273 bp (Figure 1B). These low-quality bases were also trimmed to ensure accurate results.

The sequencing coverage was lower in several exons than in the other exons. To explain this result, we analyzed the GC content and found that the regions with lower coverage (less than 80%) had excessively low (less than 40%) or high (more than 60%) GC content. The imbalanced GC content may explain why the multiplex PCR did not achieve uniform amplification of every amplicon [15]. Although the PCR conditions were optimized to amplify all the coding regions, it was difficult to avoid cross reaction because different primer pairs were mixed in one reaction. This method could have resulted in low PCR specificity when amplifying certain regions of the genes. Adjusting the maximum number of pooled samples per chip [15] and minimizing other artificial effects, such as unequal loading, would reduce non-specific amplification and false-negative variant detection.

PGMs have been reported to be biased toward the detection of indels; 92.1% of the novel indels called using the PGM in this study were of this class (Figure S2), and most of these indels could not be confirmed by Sanger sequencing. Although some of these indels fell within the context of homopolymers or tri-nucleotide repeats as observed in previous publications [16], [21], others were randomly distributed throughout the sequenced genes. To accurately identify the indels, we used two different analysis software packages to call indels in this study. The overlapping results were validated by Sanger sequencing, and 50% (three indels) were confirmed (Table S5).

Another advantage of using the PGM sequencer is the cost and time efficiency of the workflow, which was consistent with previous reports [15], [16]. Using the barcode system, six genes from 15 individuals can be sequenced simultaneously in two hours. Therefore, only 24 hours were required to sequence all the coding regions of these six genes from 93 individuals using a 314 or 316 chip, which greatly reduces the labor and time requirements compared to the cumbersome and lengthy Sanger sequencing method. Furthermore, with the newly developed 318 chip, more samples and regions could be sequenced in an improved 1G high-throughput setting.

In screening for actual disease-causing mutations, complete core families have an advantage over non-core families. The presence of data from both unaffected parents aids in the removal of unrelated SNVs when following the autosomal recessive inheritance principles. The removal rate was 73% (76/104) in the core families, but only 10% (4/40) in the non-core families. Therefore, when dealing with massive data produced by NGS for the study of monogenic diseases, collecting DNA from a complete pedigree would help to remove false-positive results and identify potential disease-causing changes.

In this study, in addition to the identification of 23 known mutations, we found six novel mutations, including five point mutations and one novel short deletion, that were supported by high read quality and depth and were confirmed by Sanger sequencing. Among these novel mutations, three novel missense mutations (P292L and I324N in the *PAH* gene and K91R in the *PTS* gene) and one nonsense mutation (Y414X), were located in the enzymes' catalytic domains and may affect protein function; however, further *in vitro* functional analysis is necessary to support this hypothesis. We also found one frame-shift mutation (N223fsdelC for the deletion of c.669delC) that result in the premature termination of protein translation. This terminated PAH protein would lack part of the catalytic domain and the entire tetramerization domain [1]. Thus, this mutation is highly likely to be a disease-causing mutation. Moreover, the IVS10-13T>G mutation is located in a region related to intron 10 splicing. Mutations within this region, such as IVS10-11G>A and IVS10-13T>G, have been suggested in PAHdb to be splice mutations. A functional study of the IVS10-11G>A mutation was performed *in vitro* and demonstrated that the mutant protein was functionally dead [22]. Despite these supportive observations, further analysis, such as minigene construction, is essential to validate the functional effect of the IVS10-13T>G mutation.

In this study, we analyzed a total of 93 samples from 35 PKU and BH4 deficiency patients and their parents (32 families), of which 29 patients were from 27 core families and six patients were from five non-core families. Using this dataset, we identified 23 known variants in the *PAH* gene that included 21 mutations from the core families and seven mutations from the non-core families (Table S5). Consistent with previous PKU analyses, several of the most prevalent mutations discovered in our analysis, R243Q, R241C, Y356X, V399V and R413P (Table 1), matched the predominant type of PKU-associated mutation found in the Asian population [1], [7]. These results suggest that these mutations are not only found in northern China but also in the central Chinese population. The migration of people from northern to southern China in recent years because of unbalanced economic development may account for the extended geographical distribution of these mutations. The mutation detection rate of 90.1% detected in this study is comparable to the 94.3% rate calculated in previous publications [1].

Despite sequencing the entire *PAH* coding region by NGS and Sanger sequencing, incomplete mutation profiles were identified in nine patients using strict criteria based on the genetic principles of autosomal recessive inheritance; six of these patients were from core families, and three were from non-core families (Table S5 with gray marks). In each of these cases, we only detected one disease-causing mutation in one allele. This observation may be due to the inability to detect additional mutations that are located in regions that were not sequenced in this study, for example, the 5′ or 3′ untranslated regions near promoters or non-coding RNA binding sites, intronic sequences far from intron/exon boundaries or large deletions. For the unsolved cases with BH4 deficiency, mutations may be located in the low coverage regions of the five genes. Possibly, other undiscovered, novel PKU-causing genes exist. Misdiagnosis may be rare, but it is also a possible explanation for the failure to identify additional disease-causing mutations in some individuals. Whole-exome sequencing will be applied in these cases after diagnosis is confirmed in future studies.

## Conclusion

This study is the first study to investigate the feasibility of using the PGM platform to study the common metabolic diseases PKU and BH4 deficiency. A detailed analysis of the mutation spectrum that was obtained by sequencing the coding regions of six genes was described. Four novel, potential disease-causing mutations were identified in the *PAH* gene. Further improvements in the PGM analysis pipeline and chip 318 would overcome the aforementioned shortcomings. As a cost- and time-effective sequencing method, the PGM would be an easy-to-use and robust platform for clinical studies of monogenic disorders.

## Supporting Information

Figure S1 **Detection of PCR products from the 108 primer pairs by agarose gel electrophoresis.** (Top) PCR products from the 108 primer pairs. (Bottom) Products from eight multiplex PCR reactions using the 108 primer pairs. I includes 15 primer pairs; II, III, IV and V include 14 different primer pairs; VI includes 23 primer pairs; VII includes 13 primer pairs; and VIII includes one primer pair.
(TIF)

Figure S2 **Distribution of insertion/deletion variant (indels) sizes detected by a PGM.**
(TIF)

Figure S3 **The result of six novel mutations in the PAH gene confirmed by Sanger sequencing.** (A), (B), (C), (D), (E) and (F) indicate c.272A>G/c.259C>T, c.669delC/c.823C>A, c.875C>T/c.728G>A, c.971T>A/c.1066-11G>A, c.1066-13T>G/c.728G>A and c.1242C>A/c.611A>G, respectively. Red indicates the novel allele.
(TIF)

Figure S4 **Evolutionary conservation analysis of the six alleles with novel mutations.** Representative reads aligned to the reference sequence (top) in exon 8 of the *PAH* gene. The boxes indicate the position with the substitution mutation (c.875C>T) as supported by 66 high-quality reads. (B) Representative reads aligned to the reference sequence (top) in exon 10 of the *PAH* gene. The boxes indicate the position with the substitution mutation (c.971T>A) as supported by 445 high-quality reads. (C) Evolutionary conservation analysis of the sequence fragment with a variant (p.P292L) in the *PAH* gene from seven species by ClustalW alignment. (D) Evolutionary conservation analysis of the sequence fragment with a variant (p.I324N) in the *PAH* gene from seven species by ClustalW alignment.
(TIF)

Figure S5 **Venn diagrams of the number of indels detected by the two algorithms.**
(TIF)

Table S1 **List of primers in this study.**
(XLSX)

Table S2 **Overview of PGM sequencing performance in all samples.**
(XLSX)

Table S3 **The relationship between mean depth and mean coverage.**
(XLSX)

Table S4 **List of potential variants in 93 samples.**
(XLSX)

Table S5 **Phenotype and genotype in 32 kindred including 35 patients.**
(XLSX)
