# Supplementary figures and images for: Mutation Spectrum of Six Genes in Chinese Phenylketonuria Patients Obtained through Next-Generation Sequencing
Source: PLoS One. 2014 Apr 4;9(4):e94100. doi: 10.1371/journal.pone.0094100 (PMC3976377; doi:10.1371/journal.pone.0094100)

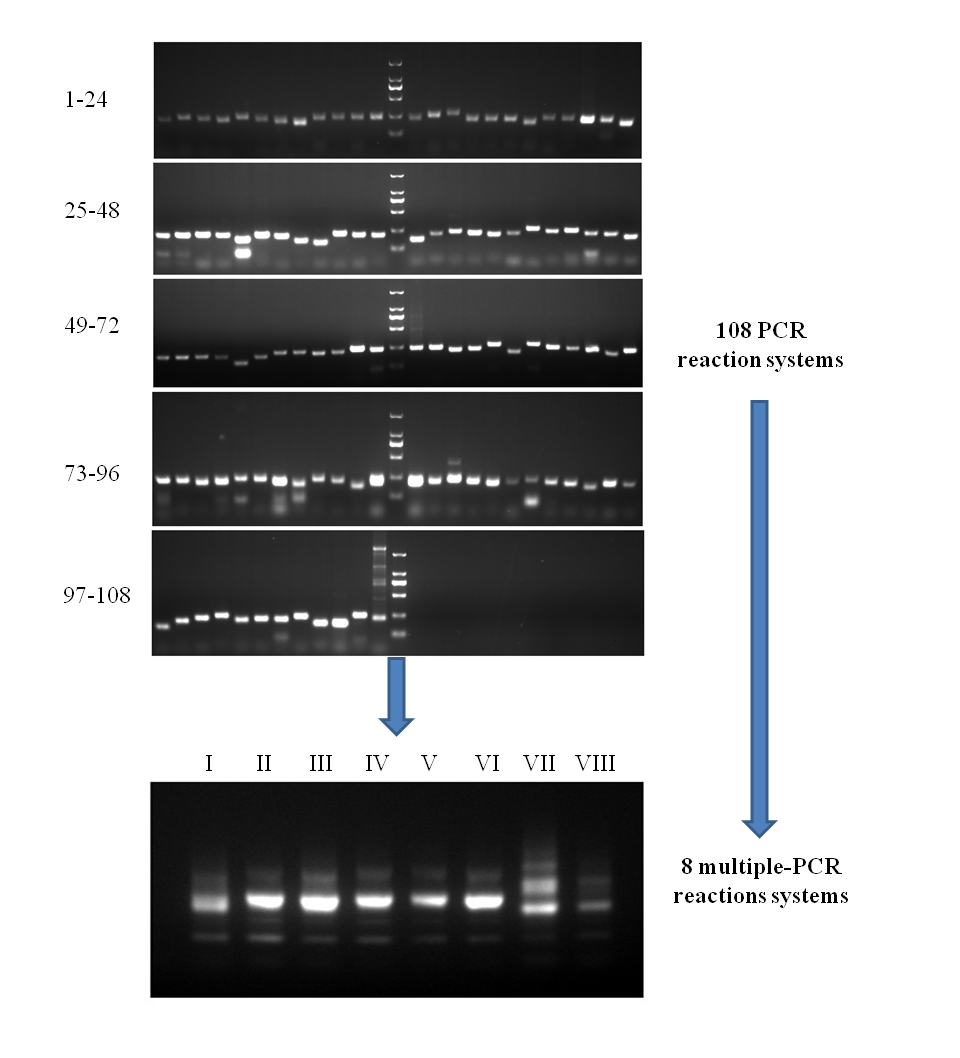

Supplement: Figure S1 — Detection of PCR products from the 108 primer pairs by agarose gel electrophoresis. (Top) PCR products from the 108 primer pairs. (Bottom) Products from eight multiplex PCR reactions using the 108 primer pairs. I includes 15 primer pairs; II, III, IV and V include 14 different primer pairs; VI includes 23 primer pairs; VII includes 13 primer pairs; and VIII includes one primer pair. (TIF) [file pone.0094100.s001.tif]

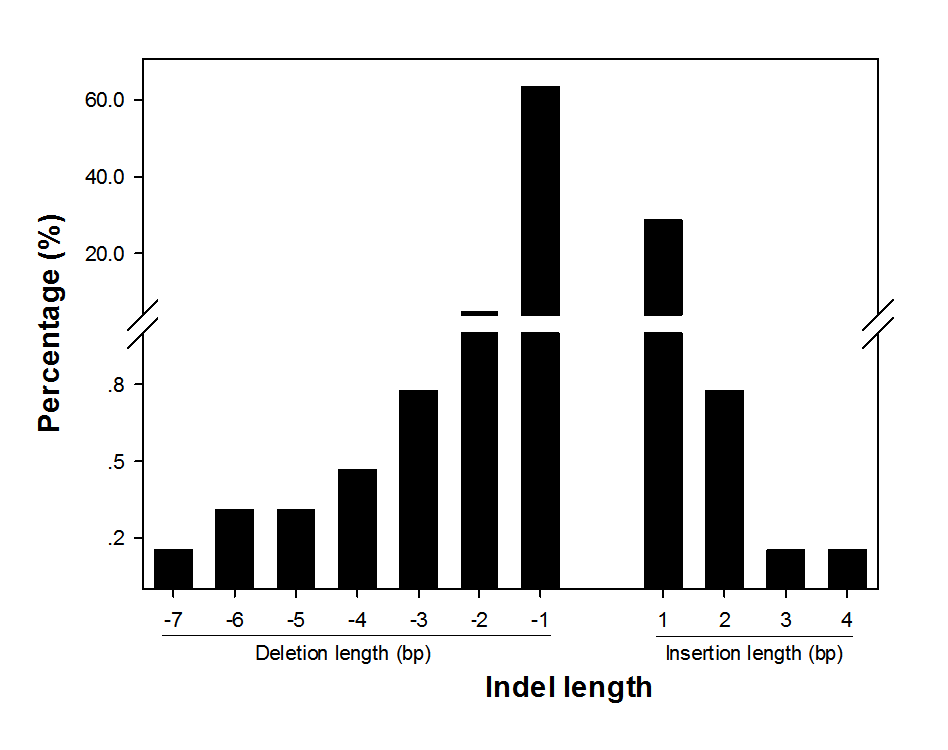

Supplement: Figure S2 — Distribution of insertion/deletion variant (indels) sizes detected by a PGM. (TIF) [file pone.0094100.s002.tif]

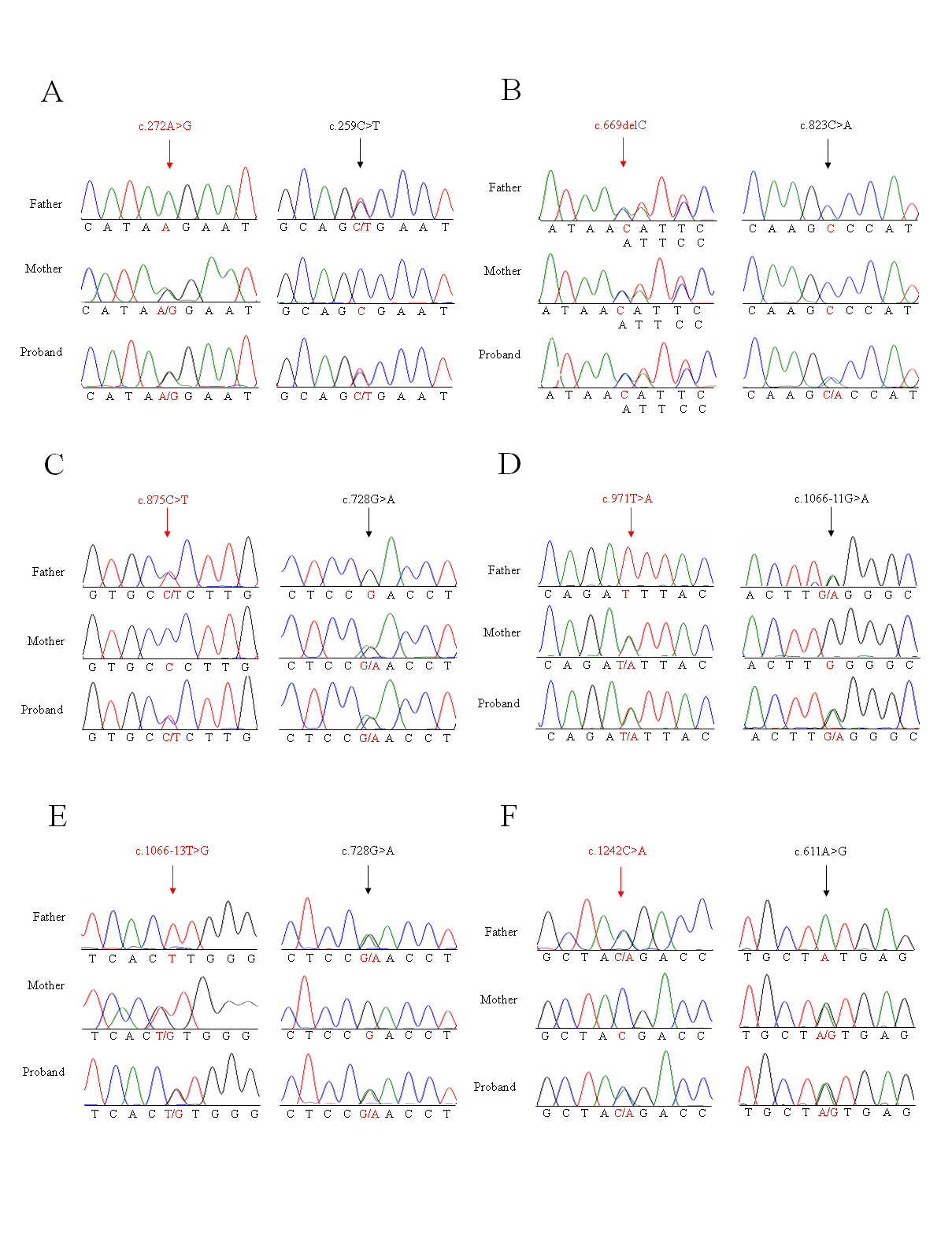

Supplement: Figure S3 — The result of six novel mutations in the PAH gene confirmed by Sanger sequencing. (A), (B), (C), (D), (E) and (F) indicate c.272A>G/c.259C>T, c.669delC/c.823C>A, c.875C>T/c.728G>A, c.971T>A/c.1066-11G>A, c.1066-13T>G/c.728G>A and c.1242C>A/c.611A>G, respectively. Red indicates the novel allele. (TIF) [file pone.0094100.s003.tif]

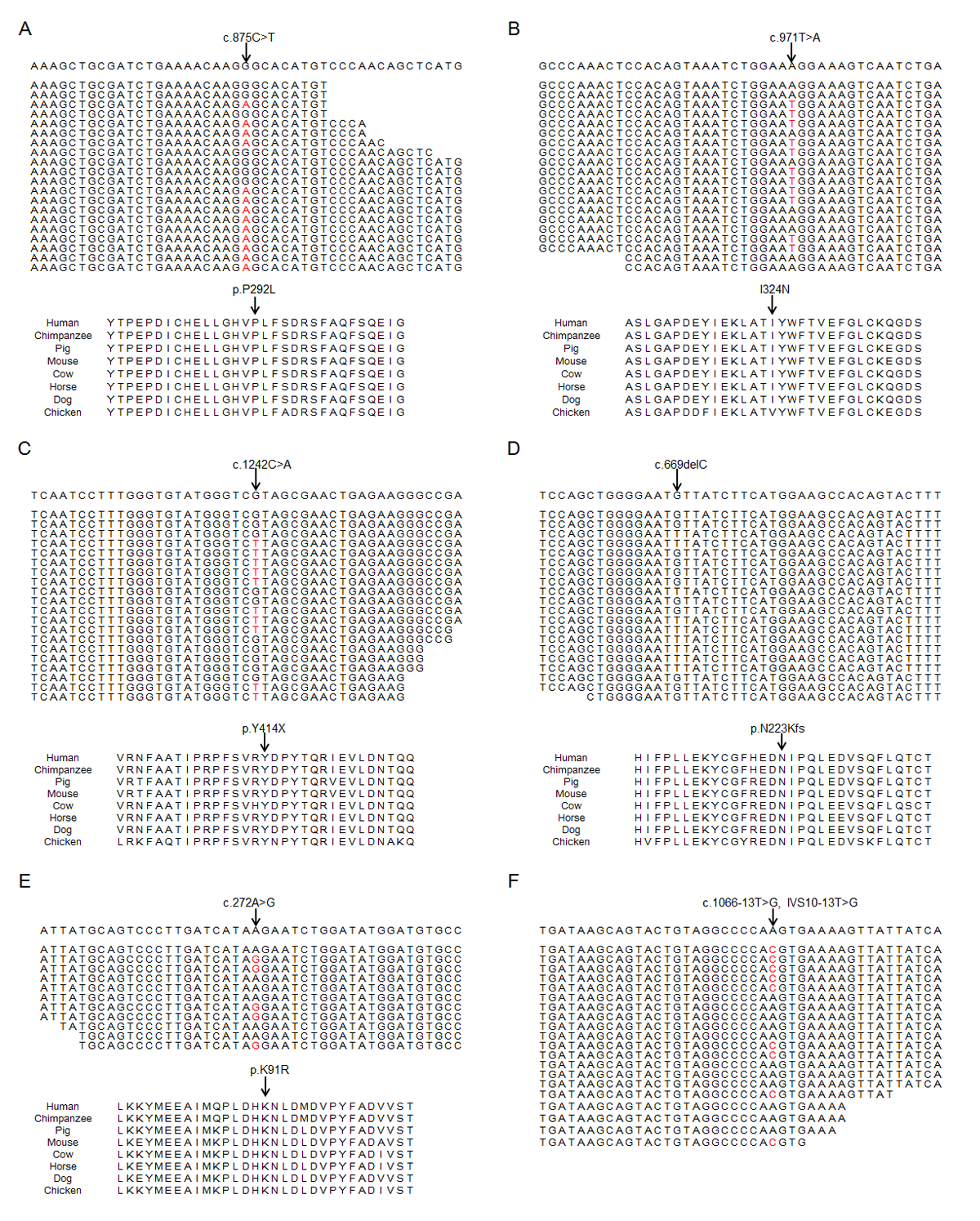

Supplement: Figure S4 — Evolutionary conservation analysis of the six alleles with novel mutations. Representative reads aligned to the reference sequence (top) in exon 8 of the PAH gene. The boxes indicate the position with the substitution mutation (c.875C>T) as supported by 66 high-quality reads. (B) Representative reads aligned to the reference sequence (top) in exon 10 of the PAH gene. The boxes indicate the position with the substitution mutation (c.971T>A) as supported by 445 high-quality reads. (C) Evolutionary conservation analysis of the sequence fragment with a variant (p.P292L) in the PAH gene from seven species by ClustalW alignment. (D) Evolutionary conservation analysis of the sequence fragment with a variant (p.I324N) in the PAH gene from seven species by ClustalW alignment. (TIF) [file pone.0094100.s004.tif]

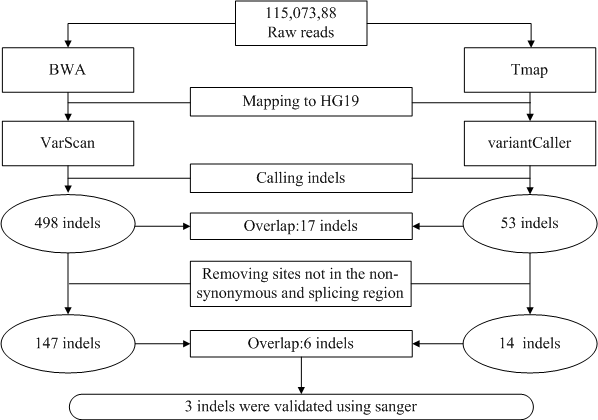

Supplement: Figure S5 — Venn diagrams of the number of indels detected by the two algorithms. (TIF) [file pone.0094100.s005.tif]
